# PIP5K1α is Required for Promoting Tumor Progression in Castration-Resistant Prostate Cancer

**DOI:** 10.3389/fcell.2022.798590

**Published:** 2022-03-21

**Authors:** Tianyan Wang, Martuza Sarwar, Jonathan B Whitchurch, Hilary M Collins, Tami Green, Julius Semenas, Amjad Ali, Christopher J Roberts, Ryan D Morris, Madlen Hubert, Sa Chen, Zahra El-Schich, Anette G Wingren, Thomas Grundström, Richard Lundmark, Nigel P Mongan, Lena Gunhaga, David M Heery, Jenny L Persson

**Affiliations:** ^1^ Department of Molecular Biology, Umeå University, Umeå, Sweden; ^2^ School of Pharmacy, University of Nottingham, Nottingham, United Kingdom; ^3^ Umeå Centre for Molecular Medicine (UCMM), Umeå University, Umeå, Sweden; ^4^ Department of Integrative Medical Biology (IMB), Umeå University, Umeå, Sweden; ^5^ Department of Pharmacy, Uppsala University, Uppsala, Sweden; ^6^ Department of Medical Biosciences, Umeå University, Umeå, Sweden; ^7^ Department of Biomedical Science, Malmö University, Malmö, Sweden; ^8^ School of Veterinary Medicine and Science, University of Nottingham, Nottingham, United Kingdom; ^9^ Department of Pharmacology, Weill Cornell Medicine, New York, NY, United States; ^10^ Department of Translational Medicine, Lund University, Clinical Research Centre in Malmö, Malmö, Sweden

**Keywords:** castration-resistant prostate cancer (CRPC), phosphatidylinositol 4-phosphate 5 kinase (PIP5K1α), matrix metalloproteinases 9 (MMP9) PIP5K1α, targeted therapy, androgen receptor (AR), cyclin-dependent kinase (CDK)

## Abstract

PIP5K1α has emerged as a promising drug target for the treatment of castration-resistant prostate cancer (CRPC), as it acts upstream of the PI3K/AKT signaling pathway to promote prostate cancer (PCa) growth, survival and invasion. However, little is known of the molecular actions of PIP5K1α in this process. Here, we show that siRNA-mediated knockdown of PIP5K1α and blockade of PIP5K1α action using its small molecule inhibitor ISA-2011B suppress growth and invasion of CRPC cells. We demonstrate that targeted deletion of the N-terminal domain of PIP5K1α in CRPC cells results in reduced growth and migratory ability of cancer cells. Further, the xenograft tumors lacking the N-terminal domain of PIP5K1α exhibited reduced tumor growth and aggressiveness in xenograft mice as compared to that of controls. The N-terminal domain of PIP5K1α is required for regulation of mRNA expression and protein stability of PIP5K1α. This suggests that the expression and oncogenic activity of PIP5K1α are in part dependent on its N-terminal domain. We further show that PIP5K1α acts as an upstream regulator of the androgen receptor (AR) and AR target genes including CDK1 and MMP9 that are key factors promoting growth, survival and invasion of PCa cells. ISA-2011B exhibited a significant inhibitory effect on AR target genes including CDK1 and MMP9 in CRPC cells with wild-type PIP5K1α and in CRPC cells lacking the N-terminal domain of PIP5K1α. These results indicate that the growth of PIP5K1α-dependent tumors is in part dependent on the integrity of the N-terminal sequence of this kinase. Our study identifies a novel functional mechanism involving PIP5K1α, confirming that PIP5K1α is an intriguing target for cancer treatment, especially for treatment of CRPC.

## Introduction

Prostate cancer (PCa) at the advanced stages often progress to a state known as castration-resistant prostate cancer (CRPC). Patients with CRPC will inevitably develop metastatic diseases especially bone metastasis. Currently, no effective therapeutic agents are available for targeting metastatic CRPC (Semenas et al., 2012; Semenas et al., 2013; Mateo et al., 2020). Thus, metastatic CRPC is an incurable disease with poor prognosis, and remains a major clinical challenge. During the development of CRPC, elevated level of androgen receptor (AR) is required and sufficient to confer androgen sensitive cells to castration resistant phenotypes (Viswanathan et al., 2018). Modulated expression of co-factors of AR is one of the mechanisms that cause altered AR expression, leading to increased survival and invasiveness of PCa under the castration-resistant condition (Feldman and Feldman, 2001). Better understanding of the AR-dependent mechanisms including: 1) constitutively active AR and AR co-factors, 2) inappropriate restoration of AR and AR co-factors, or 3) indirect AR activation, is important for designing new therapeutic interventions to treat metastatic CRPC.

We have previously identified a lipid kinase phosphatidylinositol 4-phosphate 5 kinase (PIP5K1α) as an important co-factor of AR to activate transcription of AR target genes for prostate cancer cell proliferation and survival (Larsson et al., 2020). Further, PIP5K1α acts together with matrix metalloproteinases 9 (MMP9) directly on AR to cooperatively promote angiogenesis and invasiveness of PCa (Mandel et al., 2018; Larsson et al., 2020). A previous study of ours using large patient cohorts has shown that PIP5K1α is highly expressed in primary PCa and is associated with poor PCa patient outcome (Semenas et al., 2014; Mandel et al., 2018). Moreover, elevated level of PIP5K1α significantly correlates with AR in primary PCa and metastatic lesions (Semenas et al., 2014; Sarwar et al., 2016). Overexpression of PIP5K1α promotes tumor growth and invasiveness by increasing the activity of PI3K/AKT in mouse xenograft models (Sarwar et al., 2016; Sarwar et al., 2019). Abnormal expression of AR and PIP5K1α/AKT pathways cooperatively contribute to growth, survival and invasiveness in various types of metastatic cancer (Shaw and Cantley, 2006; Janku et al., 2018). Given that PIP5K1α is a predominant kinase to produce phosphatidylinositol 4,5-trisphosphate PI(4,5)P2 (PIP2) for the activation of PI3K/AKT pathways (Loijens and Anderson, 1996; Barrero-Villar et al., 2008; van den Bout and Divecha, 2009), this implicates that PIP5K1α is a key player of the signaling cascades that promote cancer cell proliferation, survival and invasiveness. However, what specific region(s) of PIP5K1α are required to promote these biological events is not known.

It is an unmet need to develop novel therapeutic agents that can effectively target AR pathways in metastatic CRPC. Several novel approaches to inhibit interactions between AR and its co-factors by using peptidomimetics have been tested to inhibit the activity of both AR and its co-factors in PCa cell lines and xenograft mice, which show promising results. We have discovered a selective PIP5K1α inhibitor, ISA-2011B, that inhibits PIP5K1α kinase activity and blocks its downstream PI3K/AKT phosphorylation, leading to reduced growth and invasion of PCa in cell lines and xenograft mouse models (Semenas et al., 2014; Sarwar et al., 2016; Mandel et al., 2018; Karlsson et al., 2020; Larsson et al., 2020). Elevated expression of AR was significantly down-regulated in PCa cells by using this PIP5K1α inhibitor (Larsson et al., 2020). Consistently, the inhibitory effect of ISA-2011B on PCa is accompanied with its ability to significantly down-regulate the elevated expression of AR, MMP9 and VEGFR2 (Drake and Huang, 2014; Flemming, 2014; Semenas et al., 2014; Sarwar et al., 2019). Further, inhibition of PIP5K1α using ISA-2011B led to proteasome-dependent degradation of both AR and AR-V7 proteins, which sensitizes resistant cell line 22RV1 to become responsive to enzalutamide treatment (Sarwar et al., 2016). However, the mechanism underlying the interaction between PIP5K1α and AR leading to the progression of CRPC remains to be investigated (Semenas et al., 2014; Sarwar et al., 2016; Mandel et al., 2018; Karlsson et al., 2020; Larsson et al., 2020).

We elucidated the role of PIP5K1α and its molecular action in tumor growth and invasion by using C4-2 cells and DU145 cells. We characterized a gene edited CRPC cell line C4-2 that expressed an N-terminally deleted PIP5K1α. We elucidated the role of the N-terminal domain of PIP5K1α in growth, survival and invasion of CRPC in *in vitro* and *in vivo* models. We further investigated the underlying molecular mechanisms associated with the full-length PIP5K1α and its N-terminal sub-domain in regulation of AR, CDK1, MMP9 and their downstream factors that are involved in growth, survival and invasion of CRPC cells. We finally confirmed that PIP5K1α is an intriguing drug target, and its inhibitor ISA-2011B has a great potential as a targeted drug candidate for the treatment of CRPC.

## Materials and Methods

### Cell Lines

LNCaP C4-2 (RRID: CVCL_4782) cells (C4-2), and LNCaP C4-2 SG (C4-2 SG) and LNCaP C4-2 PIP5K1αΔN (C4-2 PIP5K1αΔN) and DU145 (RRID: CVCL_0105) were used in this study. C4-2 cell line (Cat#CRL-3314™) and DU145 cells (cat#HTB-81™) were purchased from American Type Culture Collection (Manassas, VA, Unites States). C4-2 PIP5K1αΔN was generated by applying CRISPR CAS9 nickase dual targeting of exon 1, leading to the deletion of the N-terminal 36 amino acids of human PIP5K1A. C4-2 SG is a single guide control that is wild type at the PIP5K1A locus, and was subjected to the same procedure of CRISPR-CAS9 gene editing applying a single guide control. The deletion of sequences that span the first ATG and before the second ATG of PIP5K1A, leaving the second ATG intact in C4-2 PIP5K1αΔN cells, was confirmed by the candidate gene sequencing. The generation and full characterisation of the C4-2 PIP5K1αΔN and C4-2 SG will be described elsewhere (CJR *et al.*; manuscript in preparation). Cells were maintained in RPMI-1640 (Cat# 32404014, Gibco™) medium supplemented with 10% fetal bovine serum (FBS, Cat# SV30160.03, Cytiva HyClone™), 1% penicillin-streptomycin-neomycin (PSN, Cat# 15640055, Gibco™) and 2 mM L-Glutamine (Cat#25-005-CI, Corning^®^). All cells used in the study were confirmed as mycoplasma-free.

### Treatment and Proliferation Assay

PIP5K1α inhibitor: ISA-2011B, a diketopiperazine fused C-1 indol-3-yl substituted 1,2,3,4-tetrahydroisoquinoline derivative (Semenas et al., 2014), at a final concentration of 50 µM in 0.1% DMSO was used for treatment for 48 h. For the proliferation assay, the MTS kit (Cat#G5421, Promega Biotech) was performed according to the manufacturer’s protocol. In brief, 5 × 10^3^ viable cells were seeded in 96 well plate in 100 µl of RPMI-1640 medium supplemented with 10% FBS, 1% PSN and 2 mM L-Glutamine. After 48 h, 20 µl of MTS reagent was added to the medium and incubated in the dark for a further 1 h. The colored formazan product produced by metabolically active live cells was measured by absorbance at 490 nm with the microplate reader Infinite^®^ M200 (Tecan). Data are represented as the percentage of control cells, and the error bar represents the standard error of the mean (SEM). For treatment of cells with proteasome inhibitor MG-132 (Cat#S2619, Selleckchem). In brief, 3 × 10^5^ cells were seeded in 6-well plate for 12 h before the treatment. MG-132 at dose of 1 µM in 0.1% DMSO, and 0.1% DMSO as control were used for treatment for 24 h, followed by the immunoblotting analysis.

### Migration Assay

After serum starvation, 5 × 10^4^ cells were seeded in serum-free RPMI-1640 medium in the upper chamber of *trans*-well migration chambers with 8 µm pores (Cat#353097, Falcon^®^). RPMI-media supplemented with 50% serum was used as a chemo-attractant in the bottom chamber (Cat#353504, Falcon^®^). After 16–24 h of incubation, the migrated cells were fixed with 4% paraformaldehyde and stained with either crystal violet or DAPI and calculated. Data are shown as a percentage of control migrated cells, and the error bar symbolize the standard error of the mean (SEM).

### The Chick Chorioallantoic Membrane-Delam Assay

The CAM-Delam assay was performed as recently described (Palaniappan et al., 2020). Briefly, 1 × 10^6^ cells were seeded inside silicone rings placed on the embryonic chorioallantoic membrane (CAM) of day 10 cultured fertilized white Bovan chick eggs (Strömbäcks Ägg, Vännäs, Sweden). The PIP5K1αΔN and SG cells were cultured in separate eggs for 2.5 and 3.5 days. Thereafter, the CAM with attached cells (CAM samples) were dissected out, fixed in 4% paraformaldehyde (Sigma-Aldrich) for 1 h at 4°C, cryoprotected in 25% sucrose solution for 1 h at 4°C. The CAM samples were frozen in frozen section medium (NEG-50, Thermo Fisher Scientific) and stored at −80°C until they were cryo-sectioned at 10 µm (HM 505 E, Microm).

### Mouse Models of Xenograft C4-2 SG and PIP5K1αΔN Tumors

The animal studies were approved by the Swedish Regional Ethical Animal Welfare Committee. The animal welfare and guidelines were strictly followed. Athymic NMRI nude male mice (Charles River Biotechnology, MA, Unites States) aged 6 weeks and weighing 25–29 g were used. SG cells (control) or PIP5K1αΔN were made in suspension in 1x PBS containing 50% of growth factor reduced Matrigel (Cat#354263, Corning^®^). 5 × 10^6^ cells/mouse were injected subcutaneously into each mouse. After tumors were established, tumor diameters were measured using a caliper, and tumor volume was calculated using the equation (a × b^2^/2), where a and b represent the larger and smaller diameters, respectively.

### Tumor Spheroid Assay

PCa cells were made in single-cell suspensions and 1 × 10^5^ cells were cultured in suspension in 2 ml of tumor spheroid formation medium (Cat#20141-500, promab Cancer Stem Premium™). The 35 mm ultra-low attachment polyhema-coated culture dishes (Cat#430588, Corning^®^) were used. The cells were subjected to the tumor-spheroid formation for 5–8 days and were then counted.

### Holomonitor Analysis

The digital HoloMonitor^®^ M4 live-cell imaging system and Hstudio™ M4 software (PHI AB, Lund, Sweden) were applied for measuring cell morphological changes and motility in response to drug treatment. The HoloMonitor is placed in an incubator at 37 °C with 5% CO_2._ For cell imaging, 0.3 × 10^6^ cells were plated in the 6-well plate. The images of live cells were captured every 10 min during the entire treatment period of 48 h. For spheroid imaging, the spheroids were firstly prepared as described under the former “Tumor Spheroid assay” section, then was transferred to a 96-well plate, and the image was taken by HoloMonitor.

### Immunofluorescence Analysis

PCa cells were grown on glass coverslips in phenol red-free RPMI-1640 medium for 24 h and fixed with 4% paraformaldehyde in PBS. The slides were washed in PBS twice and permeabilized in 0.5% Triton X-100 (Cat#T8787, Sigma-Aldrich) for 10 min at room temperature (RT). The slides were stained with primary antibodies at +4°C overnight. Primary antibodies including anti-PIP5K1α (Cat#15713-1-AP, Proteintech, 1:400), anti-AR (Cat#sc-7305, Santa Cruz, 1:200) were used. The secondary antibodies, including goat anti-rabbit IgG conjugated with Alexa Fluor 555 (Cat#A-21429, Invitrogen, 1:300), donkey anti-mouse IgG conjugated with Alexa Fluor 488 (Cat#A-21202, Invitrogen, 1:300), were used. The cell structure and nucleus were, respectively, highlighted by Rhodamine-conjugated phalloidin (Cat#R415, Invitrogen) and DAPI (4’,6-diamidino-2-phenylindole, dihydrochloride, Cat#A1001, PanReac AppliChem). The images were viewed and taken under a Leica Confocal fluorescent microscope (Leica SP8, Wetzlar, Germany) and software LAS X (Leica, Wetzlar, Germany) were used. For sectioned CAM-Delam samples, immunohistochemistry was performed using standard protocols (Wittmann et al., 2014). Sections were blocked in 10% FBS prior to primary antibody incubation at 4°C overnight. Primary anti-Ezrin rabbit (Santa Cruz sc-20773; 1:100) and anti-E-Cadherin mouse (DSHB #7D6, 1:50) antibodies were used. Secondary antibodies used were; anti-rabbit Cy3 (1:400, Jackson Immuno Research) and anti-mouse Alexa Fluor 488 (Invitrogen, 1:400) together with DAPI (Sigma-Aldrich, 1:400). Sections were mounted with fluorescence mounting medium (Allent Technologies). Stained sections were photographed using an epifluorescence microscope (Nikon Eclipse, E800) equipped with a digital camera (Nikon DS-Ri1) and images were processed with Photoshop CC 2019 (Adobe, San Jose, CA, Unites States).

### Subcellular Fractionation, Immunoprecipitation Assays, and Immunoblotting

Subcellular fractionation, immunoprecipitation analysis, and immunoblotting were performed as described previously (Semenas et al., 2014). Briefly, protein from different subcellular fractions was prepared by using Subcellular Protein Fractionation Kit for Cultured Cells (Cat#78840, Thermo Scientific™) according to the manufacturer’s protocol. For immunoprecipitation analysis, the Protein G Sepharose™ 4 Fast Flow beads (Cat#17-0618-01, GE Healthcare) and anti-PIP5K1α antibody (Cat#15713-1-AP, Proteintech) were applied to pull down PIP5K1a from the protein lysates. The rabbit IgG isotype (Cat#02-6102, Invitrogen) was used as a control. For immunoblotting, antibodies against β-tubulin (Cat#075K4875, Sigma-Aldrich) and Lamin B (Cat#sc-6216, Santa Cruz) were used, respectively, as controls for cytoplasmic and nuclear fractions. The signal was captured and documented with the Proxima C16 Phi+ imaging system (Isogen Lifescience). Densitometric quantification of immunoblots was performed by the software ImageJ 1.50i Software (NIH, Baltimore, MD, Unites States) and represented as fold change relative to control and was normalized relative to actin or GAPDH bands.

### Quantitative RT-PCR

Total RNA and cDNA were prepared respectively by the RNeasy Mini Kit (Cat#74104, QIAGEN) and the RevertAid First Strand cDNA Synthesis Kit (Cat#K1622, Thermo Scientific). The synthesized cDNA was used as a template for PCR amplification of PIP5K1A (Forward primer: AGA AGA TTC CCT GCG TTC ACC, Reverse primer: GAT CTA GAC TAT GGG TGA ACT CTG ACT CTG) and GAPDH (Forward primer: AAC AGC GAC ACC CAC TCC TC, Reverse primer: GGA GGG GAG ATT CAG TGT GGT) by using the Phire Hot Start II DNA Polymerase Kit (Cat#F122S, Thermo Scientific). The PCR product was analyzed by gel electrophoresis in 1% agarose. The signal was captured and documented with the Proxima C16 Phi+ imaging system (Isogen Lifescience). Densitometric quantification was performed by the software ImageJ 1.50i Software (NIH, Baltimore, MD, Unites States) and represented as fold change relative to control and was normalized relative to GAPDH.

### siRNA Mediated Gene Knockdown

In PIP5K1α knockdown experiment, C4-2 cells or DU145 cells were transfected with PIP5K1A-siRNA (Cat#L-004780-00-0020, Dharmacon Inc.) or non-targeting control siRNA (Cat#D-001810-10-20, Dharmacon Inc.) respectively by using TransIT-TKO^®^ kit (Cat# MIR2150, Mirus Bio LLC) or Lipofectamine™ 2000 (Cat#11668019, Invitrogen™) according to the manufacturer’s instructions. The cells were collected after 48 h transfection.

### Cell Cycle Analysis

After 48 h of siRNA transfection, the cell was collected and fixed by ice-cold 70% ethanol. The cell was then treated by 100 μg/ml of PureLink™ RNase A (Cat#12091021, Invitrogen™) and stained by 50 μg/ml of Propidium Iodide (Cat#P3566, Invitrogen™). After 15 min of incubation at the room temperature, the cells was analysed by the ZE5 Cell Analyzer (BIO-RAD). The data was analyzed by FCS Express Flow Cytometry Software (De Novo Software).

### Statistical Analysis

Student’s *t*-tests was performed. All statistical Student’s *t*-tests were two tailed, and *p* values less than 0.05 were considered to be statistically significant. Data presented is representative of at least three independent experiments. The mean is the average value of all samples. The standard error (SE) is an indication of the variability of all samples.

## Results

### The Molecular and Biological Effects of PIP5K1α and Its Association With AR-Mediated Pathway in CRPC Cells

**FIGURE 1:**
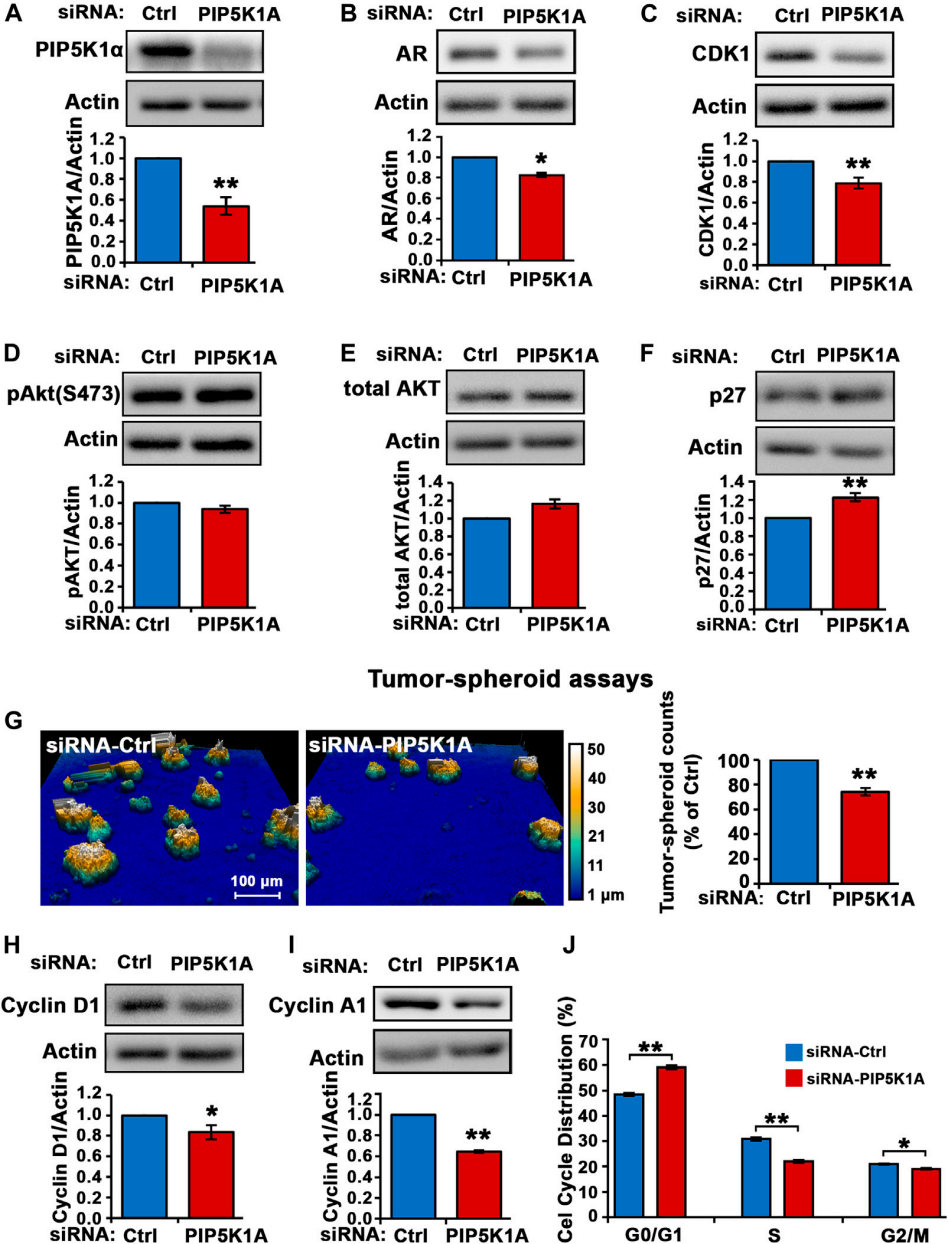
The effect of PIP5K1α inhibition on PIP5K1α-associated pathways in C4-2 cells. **(A)** PIP5K1α was silenced by transfecting C4-2 cells with control-siRNA (Ctrl-siRNA) or PIP5K1A-siRNA. Immunoblot analysis of PIP5K1α expression in Ctrl-siRNA or PIP5K1A-siRNA transfected cells is shown. Quantifications of the immunoblots for PIP5K1α expression are shown. **(B,C)** The effect of knockdown of PIP5K1α on AR and CDK1 was detected using immunoblot analysis. **(D,E,F)** Immunoblot analysis of pAKT, total AKT and P27 in Ctrl-siRNA or PIP5K1A-siRNA transfected cells is shown. **(G)** The digital Holomonitor M4 live-cell imaging system was applied to capture the tumor spheroids derived from C4-2 cells transfected with Ctrl-siRNA or PIP5K1A-siRNA. Representative images of tumor spheroids are shown in the left panel. The quantification of the counts of tumor spheroids from each group is shown in the right panel. **(H,I)** Immunoblot analysis shows the expression of cyclin D1 and cyclin A1 in Ctrl-siRNA or PIP5K1A-siRNA transfected cells. **(J)** Cell cycle distributions of C4-2 cells transfected with CtrlsiRNA or PIP5K1A-siRNA were analysed by flow cytometry. Different cell cycle phases: G0/G1, S and G2/M are indicated. Data in this figure is presented as the average of three independent experiments (±SE). Student’s *t*-test was used in the analysis. *p < 0.05 or **p < 0.01 are indicated.

The role and molecular action of PIP5K1α in CRPC remains unknown. To this end, we assessed the role of PIP5K1α in the regulation of its downstream AR and its associated key factors that contribute to the survival and progression of CRPC. Silencing of PIP5K1α in C4-2 cells was achieved by using siRNA-mediated knockdown. Immunoblot analysis confirmed the reduction of PIP5K1α expression (by 46%) after targeted siRNA treatment, compared to control (scrambled siRNA) (siRNA control cells mean = 0.35, 95% CI: 0.29–0.41; siPIP5K1α cells mean = 0.19, difference = 0.16, 95% CI: 0.13–0.26, *p* = 0.0014) (Figure 1A). Silencing of PIP5K1α resulted in a significant decrease in AR expression (siRNA control cells mean = 4.35, 95% CI: 3.65–5.05; siPIP5K1α cells mean = 3.74, difference = 0.60, 95% CI: 3.42–4.06, *p* = 0.0197, Figure 1B). Consistent with the effects observed for AR, knockdown of PIP5K1α in C4-2 cells also resulted in a significant decrease in expression of the AR downstream target CDK1 (siRNA control cells mean = 1.53, 95% CI: 1.25–1.81; siPIP5K1α cells mean = 1.20, difference = 0.33, 95% CI: 0.94–1.47, *p* = 0.0058, Figure 1C). Thus, this data suggests that PIP5K1α plays an important role in regulation of AR pathways in CRPC cells. Although silencing of PIP5K1α did not led to a significant decrease in expression of phosphorylated AKT (*p* = 0.0848, Figures 1D,E), expression of P27, a key downstream factor of AKT pathway, was significantly increased in siPIP5K1α cells compared with that of siRNA control cells (siRNA control cells mean = 0.87, 95% CI:.0.82-0.92; siPIP5K1α cells mean = 1.18, difference = 0.20, 95% CI: 0.95-1.18, *p* = 0.0023. Figure 1F). This suggests that silencing of PIP5K1α led to activation of P27, probably through blocking activity of AKT pathway. Next, we examined the functional consequences of silencing PIP5K1α *on growth of CRPC cells by using tumor-spheroid formation assays.* The number of tumor-spheroids derived from siPIP5K1α cells was significantly reduced, i.e. a 30% decrease relative to siRNA controls (siRNA control cells mean = 3,547, 95% CI: 3,181–3,913; siPIP5K1α cells mean = 2,467, difference = 1,080, 95% CI: 2,263–2,671, *p* = 0.0072, Figure 1G). Consistent with this, expression of the key regulators for proliferation and invasion including cyclin D1 (siRNA control cells mean = 0.28, 95% CI: 0.24–0.33; siPIP5K1α cells mean = 0.21, difference = 0.07, 95% CI: 0.20–0.23, *p* = 0.0132, Figure 1H) and cyclin A1 (siRNA control cells mean = 1.50, 95% CI: 1.38–1.62; siPIP5K1α cells mean = 0.97, difference = 0.53, 95% CI: 0.93–1.01, *p* = 0.0012, Figure 1I) were significantly down-regulated in siPIP5K1α cells compared with that of controls. Given that inhibition of PIP5K1α via siRNA-mediated knockdown led to significant dcrease in expression of the key cell cycle regulators, we hypothesized that PIP5K1α knockdown may affect the cell cycle of C4-2 cells. We subjected siRNA control cells and siPIP5K1α cells to the cell cycle analysis. Consistent with the effects of PIP5K1α knockdown on growth of tumor spheroids and expression of the major cell cycle regulators, the G0/G1 cell in the siPIP5K1α-treated sample was significantly increased as compared with that of sicontrol cells (p < 0.0001, Figure 1J). These data suggest that PIP5K1α plays a role in cell proliferation of C4-2 cells and exerts its effect on AR and cell cycle pathways.

**FIGURE 2:**
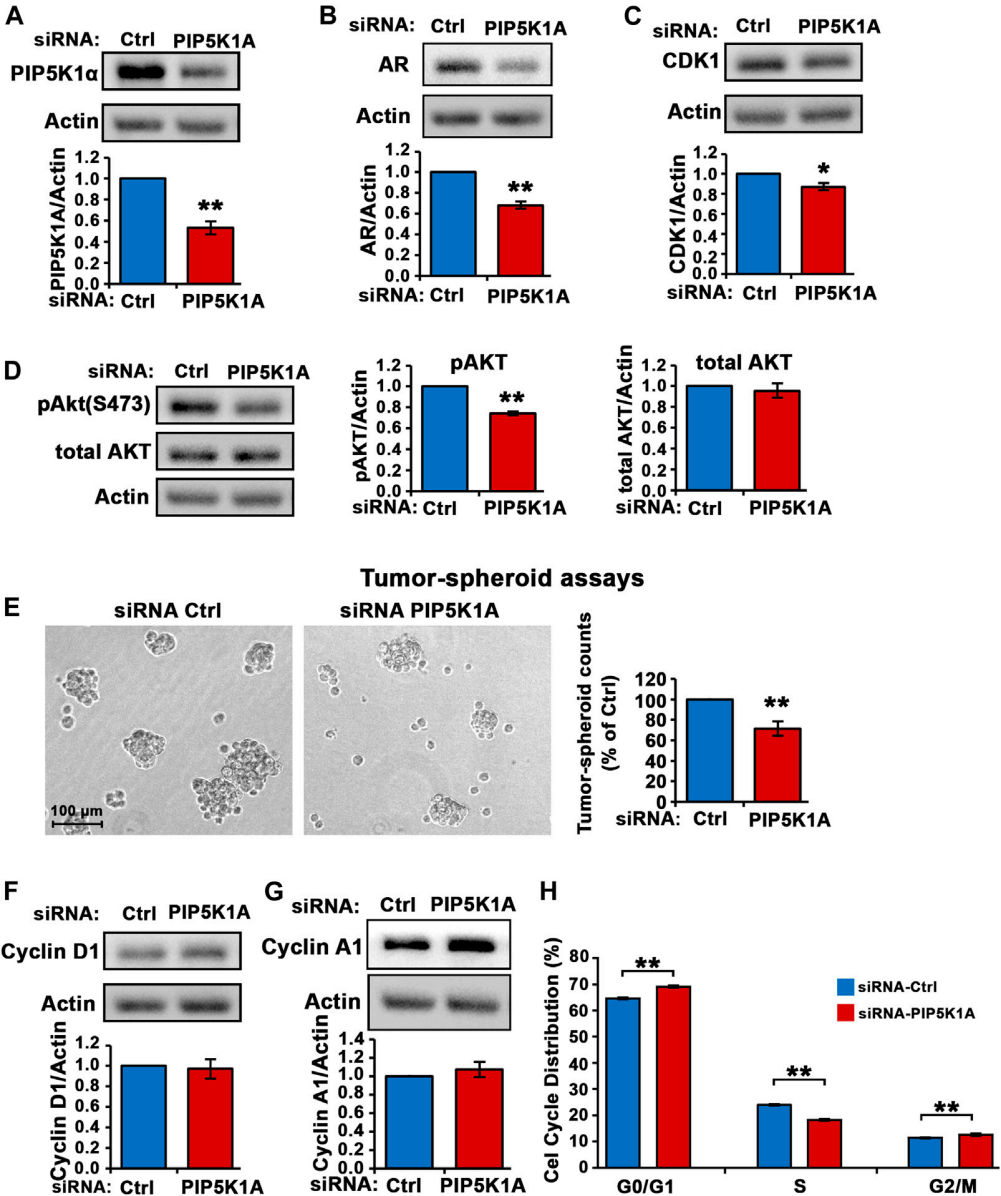
The effect of PIP5K1α inhibition on PIP5K1α-associated pathways in DU145 cells. **(A)** PIP5K1α was silenced by transfecting DU145 cells with control-siRNA (Ctrl-siRNA) or PIP5K1A-siRNA. Immunoblot analysis of PIP5K1α expression in Ctrl-siRNA or PIP5K1A-siRNA transfected cells is shown. Quantifications of the immunoblots for PIP5K1α expression are shown in the below panel. **(B,C)** Immunoblot analysis on the expression of AR and CDK1 in Ctrl-siRNA or PIP5K1A-siRNA transfected cells is shown. Quantifications of the immunoblots for AR and CDK1 expression are shown in the below panel. **(D)** Immunoblot analysis on the expression of pSer-473 AKT and total AKT in Ctrl-siRNA or PIP5K1A-siRNA transfected cells is shown. Quantifications of the immunoblots for pSer-473 AKT and total AKT expression are shown in the right panels. **(E)** The tumor spheroids derived from DU145 cells transfected with Ctrl-siRNA or PIP5K1A-siRNA. Representative images of tumor spheroids taken under the microscope are shown in the left panel. The quantification of the counts of tumor spheroids from each group is shown in the right panel. **(F,G)** Immunoblot analysis on the expression of cyclin D1 and cyclin A1 in Ctrl-siRNA or PIP5K1A-siRNA transfected cells is shown. Quantifications of the immunoblots for cyclin D1 and cyclin A1 expression are shown in the below panels. **(H)** Cell cycle distributions of Ctrl-siRNA or PIP5K1A-siRNA transfected DU145 cells were analysed by flow cytometry. Different cell cycle phases: G0/G1, S and G2/M are indicated. Data in this figure is presented as the average of three independent experiments (±SE). Student’s *t*-test was used in the analysis. *p < 0.05 or **p < 0.01 are indicated.

To confirm the role of PIP5K1α in regulation of invasive pathways responsible for progression of CRPC, another CRPC cell line expressing mutated AR, DU145, was used. The human DU145 cell line is derived from brain metastatic PCa and has high metastatic potential (Stone et al., 1978). DU145 cells were transfected with control siRNA and siRNA to PIP5K1A. Silencing of PIP5K1α in DU145 cells was achieved using siRNA-mediated knockdown. Immunoblot analysis confirmed the reduction of PIP5K1α expression (by 47%) after targeted siRNA treatment, compared to control (scrambled siRNA) (siRNA control cells mean = 1.67, 95% CI: 1.56–1.78; siPIP5K1α cells mean = 0.89, difference = 0.78, 95% CI: 0.67–1.11, *p* = 0.0016; Figure 2A). Silencing of PIP5K1α resulted in a significant decrease in AR expression (siRNA control cells mean = 1.19, 95% CI: 0.78-1.60; siPIP5K1α cells mean = 0.82, difference = 0.37, 95% CI: 0.50-1.14, *p* = 0.0007, Figure 2B). Similar to what was observed in C4-2 cells, knockdown of PIP5K1α in DU145 cells also resulted in a significant decrease in expression of CDK1 (siRNA control cells mean CDK1 = 1.07, 95% CI: 1.02–1.13; siPIP5K1α cells mean CDK1 = 0.94, difference = 0.14, 95% CI: 0.87–1.00, *p* = 0.0177, Figure 2C). Since constitutive activation of AKT play important role in invasive CRPC cells (Sarwar et al., 2016), we also examined the effect of PIP5K1α-knockdown on the expression of Ser-473 AKT in DU145 cells. Silencing of PIP5K1α resulted in a significant decrease in expression of Ser-473 AKT (for siRNA control cells, mean expression Ser-473 AKT = 1.29, 95% CI: 1.19–1.40; for siPIP5K1α cells, mean expression Ser-473 AKT = 0.96, difference = 0.33, 95% CI: 0.90–1.02 *p* = 0.0002, Figure 2D). The total AKT remained virtually unchanged. PIP5K1α acts on AR and PI3K/AKT-associated pathways in DU145 cells. We examined the functional consequences of silencing PIP5K1α *on growth of DU145 cells by using tumor-spheroid formation assays.* The number of tumor-spheroids derived from siPIP5K1α cells was significantly reduced, i.e. a 29% decrease relative to siRNA controls (siRNA control cells mean = 1,003, 95% CI: 653-1,351; siPIP5K1α cells mean = 731, difference = 272, 95% CI: 531-931, *p* = 0.0025, Figure 2E). PIP5K1α-knockdown had no significant effect on expression of cyclin D1 and cyclin A1 (for cyclin D1, *p* = 0.7781, for cyclin A1, *p* = 0.4194, Figures 2F,G). We subjected siRNA control cells and siPIP5K1α DU145 cells to the cell cycle analysis. Consistent with the effects of PIP5K1α knockdown on growth of tumor spheroids, the proportion of the siPIP5K1α cells at G0/G1 was significantly increased as compared with that of sicontrol cells (*p* < 0.0001, Figure 2H). The proportion of the siPIP5K1α cells at onset of S phase was significantly decreased as compared with that of sicontrol cells (*p* < 0.0001, Figure 2H). These data suggest that PIP5K1α is functionally important in regulation of cell cycle progression in DU145 cells.

### Characterization of PIP5K1α as a Target for its Inhibitor ISA-2011B in C4-2 Cells

**FIGURE 3:**
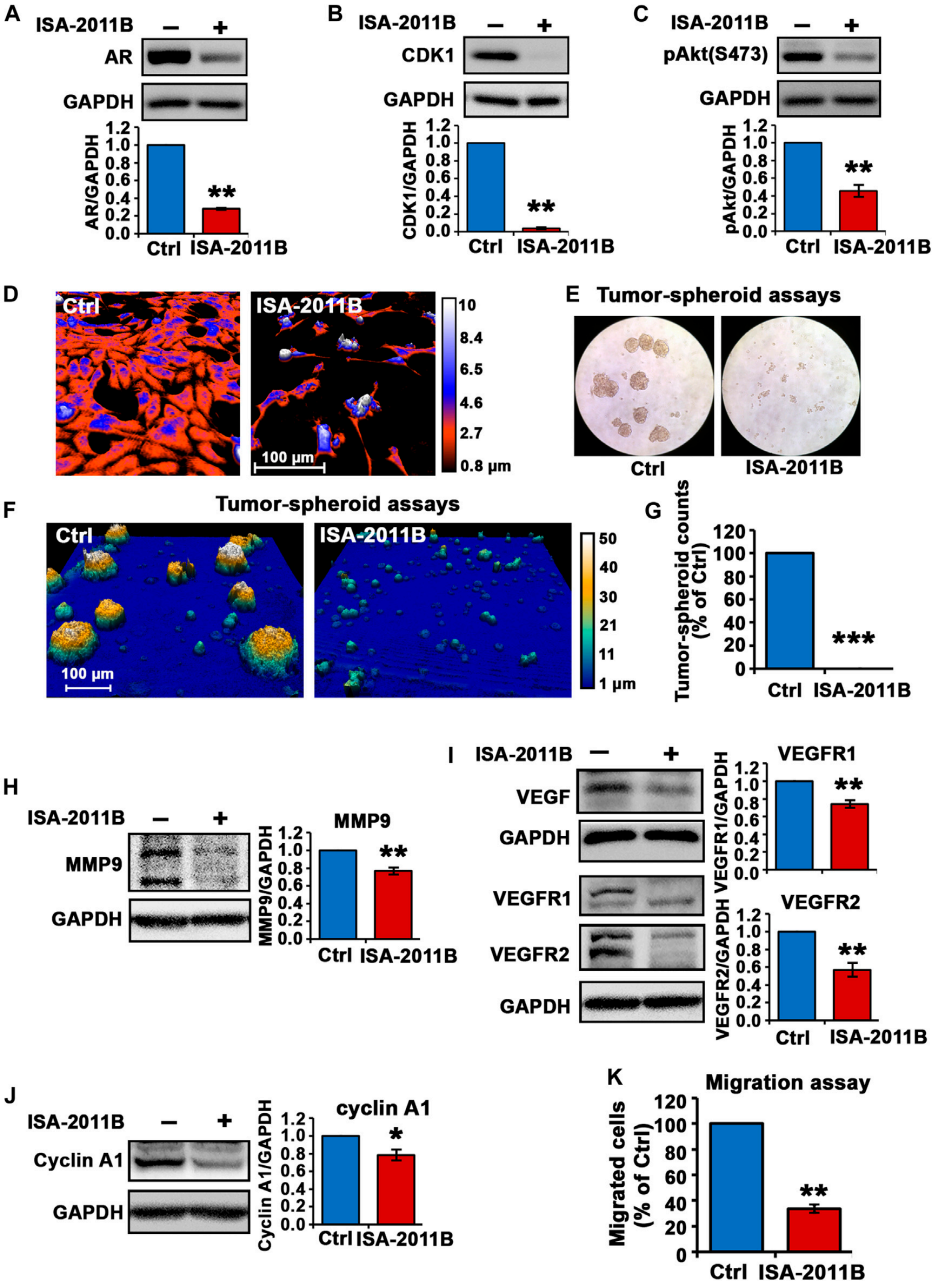
Inhibition of total PIP5K1α by its inhibitor ISA-2011B on functional pathways in C4-2 cells. **(A–C)** The effect of ISA-2011B on the expression of AR, CDK1 and pAKT in C4-2 cells was determined using immunoblot analysis. Quantifications of the immunoblots are shown in the lower panels. **(D)** Representative live-cell images from the digital Holomonitor M4 microscope shows the morphological changes in C4-2 cells that were treated with vehicle control (Ctrl) or ISA-2011B for 48 h. Representative images of tumor spheroids derived from cells treated with vehicle control (Ctrl) or ISA-2011B were taken by using a light microscope in **(E)** and the Digital Holomonitor M4 microscope **(F)**. **(G)** The counts of tumor spheroids derived from C4-2 cells treated with vehicle or ISA-2011B are shown. **(H–J)** The effect of ISA-2011B on the expression of MMP9, VEGF, VEGFR1, VEGFR2 and Cyclin A1 was determined using immunoblot analysis. Quantifications of the immunoblots are shown in the right panels. **(K)** The effect of ISA-2011B on the migratory ability of C4-2 cells was determined using migration assays. Data in this figure is presented as the average of three independent experiments (±SE). Student’s *t*-test was used in the analysis. *p < 0.05 or **p < 0.01 are indicated.

It is not known whether PIP5K1α inhibitor ISA-2011B may exert its effect on growth and/or survival of CRPC C4-2 cells, we therefore examined the molecular and biological effects of inhibition of PIP5K1α via its inhibitor ISA-2011B in C4-2 cells. ISA-2011B treatment led to a 72 and 96% decrease in expression of AR and CDK1, respectively, in C4-2 cells compared with vehicle control (for control-treated, mean AR expression = 1.37, 95% CI: 1.10-1.63; for ISA-2011B treated cell, mean AR expression = 0.39, difference = 0.98, 95% CI: 0.28-0.50, *p <* 0.001; for control-treated, mean CDK1 expression = 3.31, 95% CI: 2.81-3.82; for ISA-2011B treated, mean CDK1 = 0.08, difference = 3.23, 95% CI: -0.01-0.17, *p* < 0.001, Figures 3A,B). Moreover, ISA-2011B treatment resulted in a significant reduction in phosphorylated Ser-473 AKT, by 54% relative to the controls (For controls, mean expression Ser-473 AKT = 1.09, 95% CI: 1.00-1.18; mean expression in *ISA-2011B treated cell* = 0.49, difference = 0.61, 95% CI: 0.37-0.60, *p <* 0.001, Figure 3C).

In addition, live cell holomonitor imaging analysis showed that ISA-2011B treatment led to a dramatic reduction in cell volume and motility of C4-2 cells (Figure 3D). We next subjected C4-2 cells treated with vehicle control or ISA-2011B to tumor-spheroid assays. Strikingly, barely any tumor-spheroid formation from the C4-2 cells that were treated with ISA-2011B were observed, with a reduction in tumor-spheroid counts by 100% relative to controls (*p* < 0.001, Figures 3E–G). This was accompanied by a significant down-regulation in expression of MMP9, VEGFR1 and VEGFR2 in C4-2 cells treated with ISA-2011B compared with that of controls (for control-treated, mean MMP9 expression = 1.60, 95% CI: 1.44-1.75; for ISA-2011B treated cell, mean MMP9 expression = 1.23, difference = 0.37, 95% CI: 1.05-1.41, *p =* 0.0013; for control-treated, mean VEGFR1 expression = 10.96, 95% CI: 8.20-13.72; for ISA-2011B treated cell, mean VEGFR1 expression = 8.05, difference = 2.92, 95% CI: 6.28-9.81, *p <* 0.001; for control-treated, mean VEGFR2 expression = 12.38, 95% CI: 7.89-16.86; for ISA-2011B treated cell, mean VEGFR2 expression = 6.93, difference = 5.45, 95% CI: 4.51-9.35, *p =* 0.0017, Figures 3H,I). In addition, cyclin A1 was significantly down-regulated in ISA-2011B-treated C4-2 cells as compared with control cells (for control-treated, mean cyclin A1 expression = 9.49, 95% CI: 5.86-13.12; for ISA-2011B treated cell, mean cyclin A1 expression = 7.37, difference = 2.12, 95% CI: 4.70-10.04, *p =* 0.0131, Figure 3J). ISA-2011B also inhibited the migratory ability of C4-2 cells (for control-treated, mean migrated cell number = 88, 95% CI: 72-103; for ISA-2011B treated cell, mean migrated cell number = 30, difference = 58, 95% CI: 20-40, *p <* 0.001, Figure 3K). These data further support that the molecular and biological effects of silencing PIP5K1α using siRNA are similar to what is achieved by blockade of PIP5K1α via its inhibitor ISA-2011B.

**FIGURE 4:**
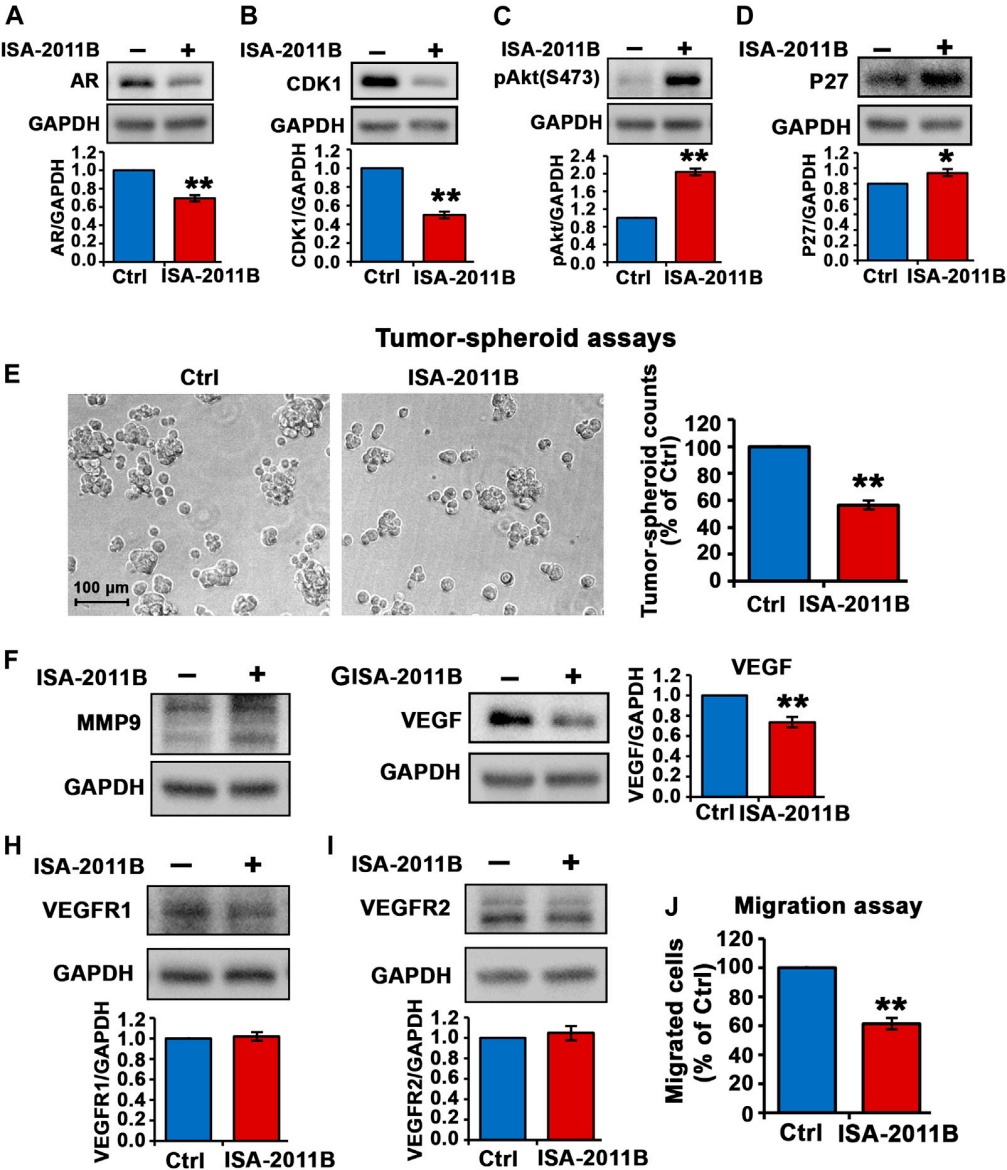
Inhibition of total PIP5K1α by its inhibitor ISA-2011B on functional pathways in DU145 cells. **(A–D)** The effect of ISA-2011B on the expression of AR, CDK1, pAKT and P27 in DU145 cells was determined using immunoblot analysis. Quantifications of the immunoblots are shown in the lower panels. **(E)** Representative images of tumor spheroids derived from DU145 cells treated with ISA-2011B or vehicle control were taken by using light microscope. The counts of tumor spheroids derived from DU145 cells treated with ISA-2011B or vehicle control are shown in the right panel. **(F–I)** The effect of ISA-2011B on expression of MMP9, VEGF, VEGFR1 and VEGFR2 in DU145 cells was determined using immunoblot analysis. Quantifications of the immunoblots are shown. **(J)** The effect of ISA-2011B on the migratory ability of DU145 cells was determined using migration assays. Data in this figure is presented as the average of three independent experiments (±SE). Student’s *t*-test was used in the analysis. *p < 0.05 or **p < 0.01 are indicated.

We followed up by examining the inhibitory effect of ISA-2011B in DU145 cells that were derived from brain metastatic PCa *with high metastatic potential. Similar to what was observed in C4-2 cells, treatment of DU145 cells with ISA-2011B led to significantly decrease in AR expression* (for control-treated, mean AR expression = 1.18, 95% CI: 1.00-1.36; for ISA-2011B treated cell, mean AR expression = 0.82, difference = 0.36, 95% CI: 0.67-0.98, *p* = 0.0005*,* Figure 4A)*.* CDK1 was significantly down-regulated in ISA-2011B-treated DU145 cells as compared with control-treated cells (for control-treated, mean CDK1 expression = 1.35, 95% CI: 1.17-1.54; for ISA-2011B treated cell, mean CDK1 expression = 0.68, difference = 0.67, 95% CI: 0.51-0.85, *p <* 0.0001, Figure 4B). ISA-2011B treatment resulted in increased expression of phosphorylated Ser-473 AKT (Figure 4C). Since P27 is a downstream protein of PI3K/AKT and also a cell cycle inhibitor, which is often down-regulated during metastatic progression of DU145 cells, we therefore examined the effect of ISA-2011B on P27 expression. P27 was significantly up-regulated in ISA-2011B-treated DU145 cells as compared with control-treated cells (for control-treated, mean P27 expression = 1.29, 95% CI: 1.21-1.37; for ISA-2011B treated cell, mean P27 expression = 1.47, difference = 0.18, 95% CI: 1.36-1.57, *p =* 0.0144, Figure 4D). This suggests that ISA-2011B treatment resulted in the activation of P27 function in DU145 cells.

We next subjected DU145 cells treated with vehicle control or ISA-2011B to tumor-spheroid assays. There was a significant decrease in the numbers of tumor-spheroids that were derived DU145 cells treated with ISA-2011B as compared with that of controls (*p* < 0.0001, Figure 4E).

Although ISA-2011B treatment did not led to down-regulation of MMP9 expression (Figure 4F, but led to significant decrease in expression of VEGF, a key angiogenic factor, in DU145 cells (for control-treated, mean VEGF expression = 1.04, 95% CI: 0.79-1.29; for ISA-2011B treated cell, mean VEGF expression = 0.77, difference = 0.27, 95% Figure 4G CI: 0.55-0.99, *p =* 0.0005, Figure 4G). ISA-2011B treatment did not have significant effect on expression of VEGFR1 and VEGFR2 in DU145 cells (Figures 4H,I). Similar to what was observed in C4-2 cells, ISA-2011B significantly inhibited the migratory ability of DU145 cells (for control-treated, mean migrated cell number = 129, 95% CI: 116-141; for ISA-2011B treated cell, mean migrated cell number = 75, difference = 54, 95% CI: 53-97, *p <* 0.0001, Figure 4J). These data confirmed that the targeted inhibition of PIP5K1α via its inhibitor ISA-2011B suppressed growth and invasion of CRPC cells. Thus, the results obtained from DU145 cells confirmed our findings in using C4-2 cells, and further provided evidence suggesting an important role of PIP5K1 in CRPC.

### PIP5K1α Stability is Mediated by Proteasome-Dependent Degradation and the N-Terminal Region is Functionally Important for Mediating PIP5K1α Expression and Protein Stability

**FIGURE 5:**
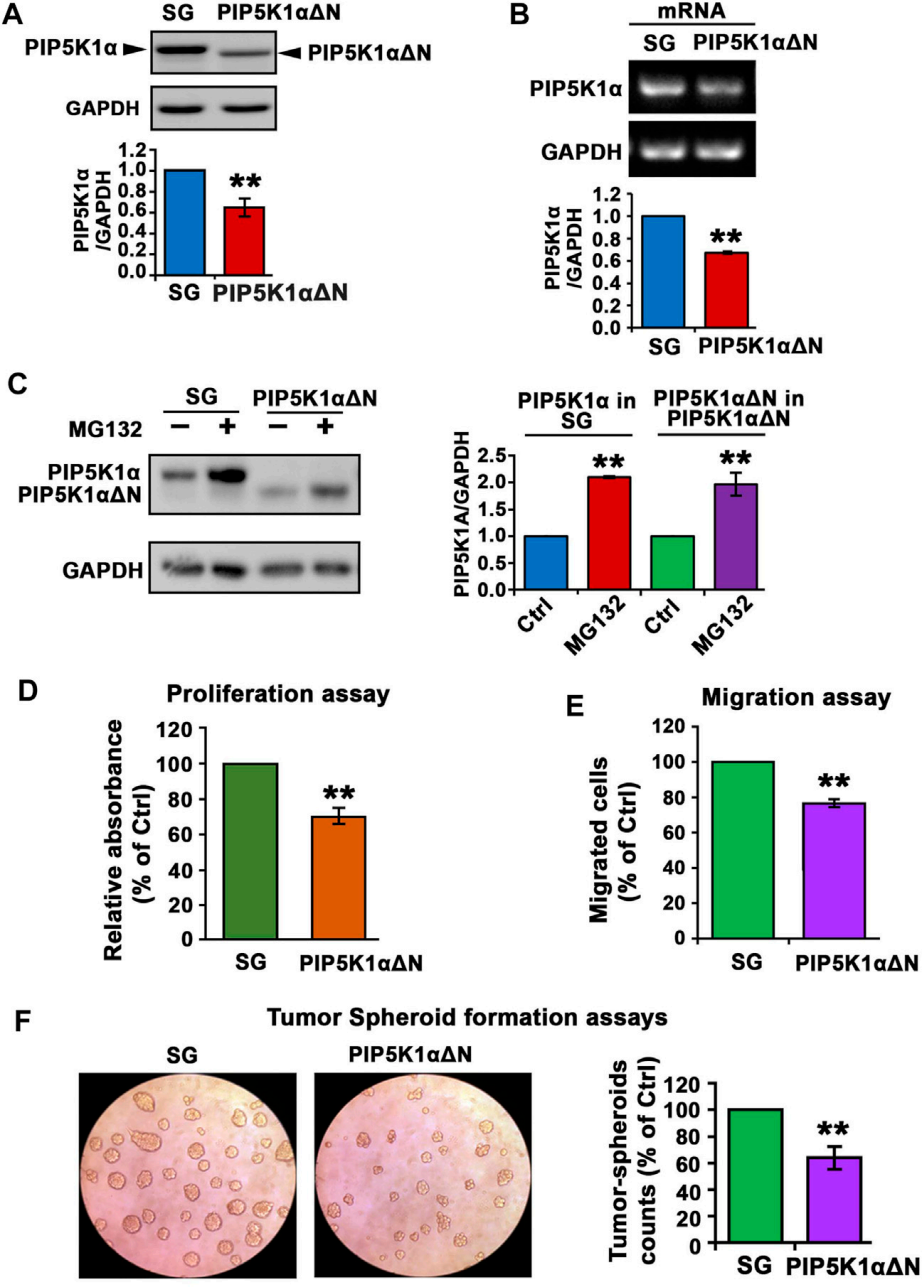
The effect of N-terminal-truncated PIP5K1α on the proliferation and migratory ability of PIP5K1αΔN C4-2 cells. **(A)** Immunoblot analysis on the expression of trunctated PIP5K1α in PIP5K1αΔN C4-2 cells (PIP5K1αΔN) or full-length PIP5K1α in SG C4-2 cells (SG) is shown. The antibody against full-length PIP5K1α was used. Quantification of the expression level of PIP5K1αΔN and PIP5K1α in the cells is shown. **(B)** Representative image of the quantitative RT-PCR to show mRNA expression of WT PIP5K1A and truncated PIP5K1A in SG cells and in PIP5K1αΔN cells, respectively. **(C)** Immunoblot analysis on the expression of PIP5K1α and PIP5K1αΔN in SG cells and PIP5K1αΔN cells that were treated with MG132 or vehicle control. Quantifications of the immunoblots for expression of PIP5K1αΔN and PIP5K1α are shown in the right panel. **(D)** Non-radioactive MTS reagent was used to measure the proliferation rate of SG cells and PIP5K1αΔN cells. **(E)** SG cells and PIP5K1αΔN cells were subjected to the trans-well Boyden chamber migration assays. The relative counts of the migrated cells are shown. **(F)** SG cells and PIP5K1αΔN cells were subjected to the tumorspheroid formation assay to assess the single-cell derived tumor-spheroid formation and growth. Representative images of tumor-spheroids derived from SG cells and PIP5K1αΔN cells are shown. The relative counts of tumor spheroids from each group are shown. Data in this figure is presented as the average of three independent experiments (±SE). Student’s *t*-test was used in the analysis. **p < 0.01 is indicated.

Based on the information from the crystal structure of PIP5K1α (Hu et al., 2015), the N-terminal sequence of PIP5K1α is important for its function. We therefore established C4-2 PIP5K1αΔN cells by using CRISPR CAS9 nickase dual targeting of exon 1 of *PIP5K1A* to delete the N-terminal 36 amino acids of PIP5K1α.C4-2 PIP5K1αΔN cells express exclusively truncated PIP5K1α encoded by the mRNA lacking the first ATG fragment, leaving the second ATG intact. RNAseq analysis of PIP5K1αΔN and control C4-2 SG cells confirmed the deletion, corresponding to a 36-amino-acid N-terminal region in PIP5K1α (data not shown). The generation and full characterisation of the C4-2 PIP5K1αΔN and control C4-2 SG will be described elsewhere (CJR *et al.*; manuscript in preparation). Immunoblot analysis confirmed the presence of the truncated protein and absence of the wild-type/full-length PIP5K1α in PIP5K1αΔN cells (Figure 5A). This data further confirmed that the targeted deletion in exon 1 of *PIP5K1A*, leading to expression of the truncated PIP5K1α protein was achieved. Interestingly, expression level of PIP5K1αΔN protein was significantly reduced by approximately 30% compared with wild-type/full-length PIP5K1α (Figure 5A). Similarly, level of mRNA encoding PIP5K1αΔN was also significantly reduced by 30% relative to that encoding for wild-type PIP5K1α (Figure 5B). This suggests that the N-terminal region is important for the regulation of PIP5K1α mRNA and protein expression in C4-2 cells.

Next, we assessed whether a decrease in PIP5K1αΔN protein expression might be in part a result of disrupted protein stabilization. To this end, we treated C4-2 PIP5K1αΔN and control C4-2 SG with MG132, a proteasome inhibitor. Expression of wild-type PIP5K1α was remarkably increased in C4-2 SG cells treated with MG132 compared with that of C4-2 SG cells treated with vehicle control (Figure 5C). This data suggests that MG132 prevented proteasome-dependent degradation of PIP5K1α. Interestingly, expression of the truncated PIP5K1α after treatment of MG132 increased to the level equivalent to that of wild-type PIP5K1α (Figure 5C). This suggests that deletion of the N-terminal region of PIP5K1α in part leads to inhibition of protein expression by disrupting its protein stabilization.

To examine the functional consequences of the N-terminal deletion in PIP5K1α on CRPC cell growth, we compared performance of PIP5K1αΔN cells and the SG (WT) control cells in proliferation assays. The proliferation rate of PIP5K1αΔN cells was found to be significantly lower compared with that of control (SG) cells (*p <* 0.001, Figure 5D). Next, we examined the functional consequence of the truncated PIP5K1α on invasive behavior of CRPC cells by subjecting PIP5K1αΔN and SG-control cells to the *in vitro* Boyden chamber migration assays. The migration rate of PIP5K1αΔN cells was significantly reduced compared to the SG control (*p* = 0.004, Figure 5E). Next, the effect of the N-terminal deletion in PIP5K1α on tumorigenic ability of CRPC cells was assessed by using three-dimensional (3-D) tumor-spheroid formation assays. The tumor spheroids derived from PIP5K1αΔN single cells were significantly less with a reduction by 36% relative to SG controls (*p* < 0.001, Figure 5F). These data suggest that loss of the N-terminal sequence of PIP5K1α hampers the tumorigenic ability of CRPC cells. Given that PIP5K1α promotes growth, survival and invasion of PCa cells (Semenas et al., 2014; Sarwar et al., 2016), our finding indicates that the N-terminal region is critical for PIP5K1α to fulfil its role in promoting growth of PCa.

**FIGURE 6:**
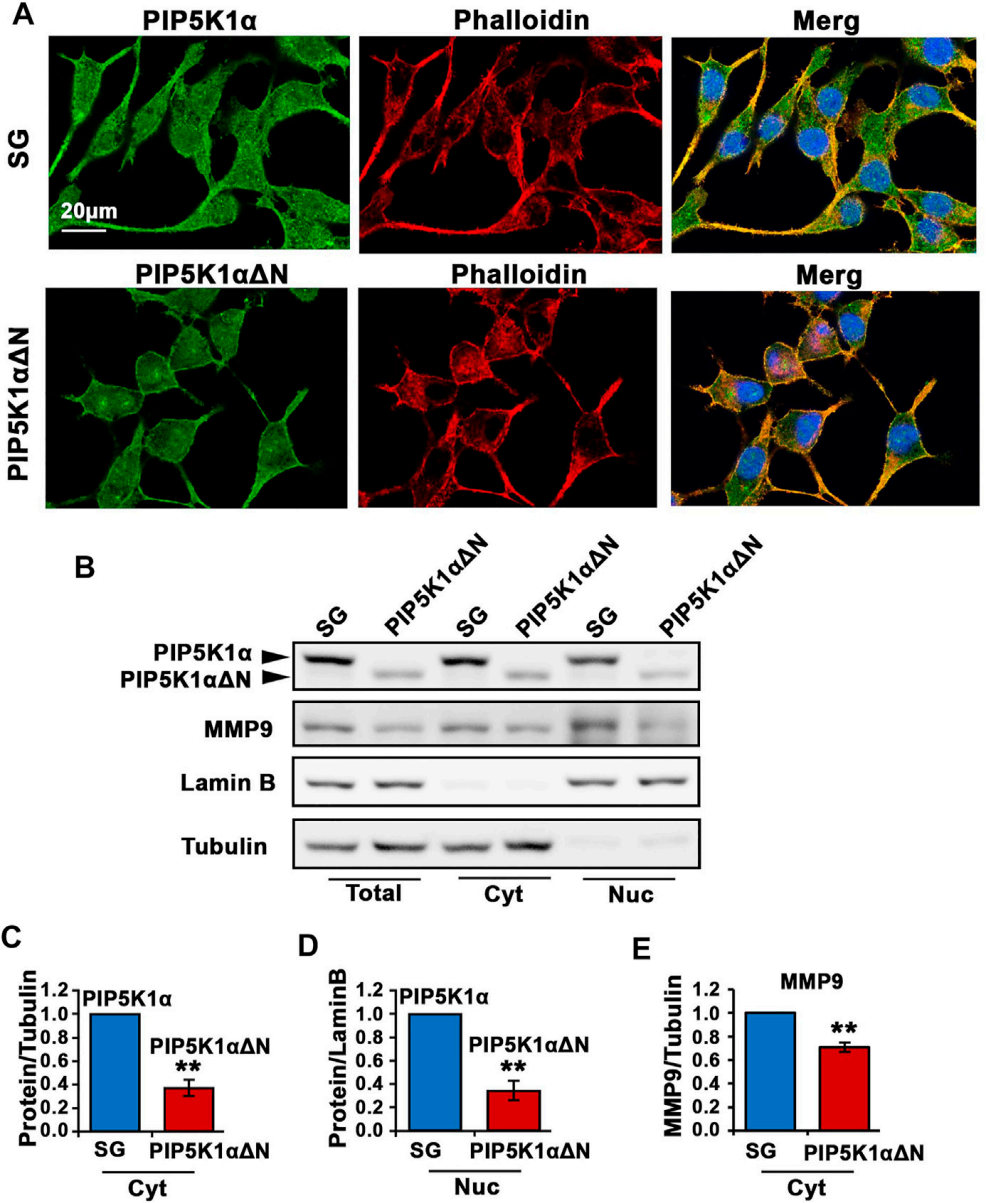
The effect of N-terminal-truncated PIP5K1α on migratory ability and subcellular components of PIP5K1αΔN cells. **(A)** Representative immunofluorescent images showed the subcellular localization of PIP5K1α and PIP5K1αΔN in green in SG and PIP5K1αΔN cells, respectively. The cells were stained by phalloidin in red. Scale bar: 20 μm. **(B)** Immunoblot analysis of subcellular localization and expression of PIP5K1α and MMP9 in SG cells and PIP5K1αΔN cells. **(C–E)** Quantifications of the immunoblots for PIP5K1α proteins and MMP9 in cytoplasmic (Cyt) and nuclear (Nuc) compartments of SG cells and PIP5K1αΔN cells are shown. Data in this figure is presented as the average of three independent experiments (±SE). Student’s *t*-test was used in the analysis. **p < 0.01 is indicated.

Immunofluorescence analysis was performed to examine subcellular localization and cellular morphology of the truncated PIP5K1α in PIP5K1αΔN cells, in comparison with SG control cells. We co-immunostained PIP5K1αΔN cells and SG control cells using an antibody against full-length PIP5K1α and phalloidin dye to highlight the cytoskeleton of the cells. Wild-type/full-length PIP5K1α was localized in both membrane/cytoplasmic and nuclear compartments of SG control cells (Figure 6A). The truncated PIP5K1α protein was also present in both subcellular compartments in PIP5K1αΔN cells. However, there were striking differences in cellular morphology and behaviour between PIP5K1αΔN cells and SG control cells. PIP5K1αΔN cells exhibited reduced cellular protrusions and disruption in cell-cell contacts as compared to that of SG cells (Figure 6A). To further assess subcellular distribution of the WT and truncated proteins we prepared subcellular fractionations of PIP5K1αΔN and SG cells followed by immunoblot analysis. We found that both nuclear and cytoplasmic levels of the truncated PIP5K1α protein in PIP5K1αΔN cells were significantly lower than that of full-length PIP5K1α in SG cells (for SG cells, mean cytoplasmic PIP5K1α expression = 2.62, 95% CI: 1.96-3.28; for PIP5K1αΔN cells, mean cytoplasmic PIP5K1α = 1.01, difference = 1.60, 95% CI: 0.43-1.60, *p* < 0.001; for SG cells, mean nuclear PIP5K1α = 0.66, 95% CI: 0.55-0.77; for PIP5K1αΔN and SG control cells cells, mean nuclear PIP5K1α = 0.23, difference = 0.43, 95% CI: 0.10-0.37, *p* = 0.0015; Figures 6B–D). Immunoblot analysis further showed that expression of MMP9, a key regulator of extracellular matrixes in PCa invasion (Sarwar et al., 2016), was significantly down-regulated in the membrane/cytoplasmic compartments of PIP5K1αΔN cells compared with that of SG controls (for SG cells, mean cytoplasmic MMP9 = 2.23, 95% CI: 1.89-2.57; for PIP5K1αΔN cells, mean cytoplasmic MMP9 = 1.57, difference = 0.66, 95% CI: 1.46-1.68, *p* = 0.0019, Figures 6B,E). These data further support that the molecular and biological effects of deletion of the N-terminal of PIP5K1α are similar to what is achieved by blockade of PIP5K1α via siRNA-knockdown or its inhibitor ISA-2011B.

### The Functional Association of PIP5K1α With AR, and the Effect of Deletion of the N-Terminal Region of PIP5K1α on Downstream Pathways

**FIGURE 7:**
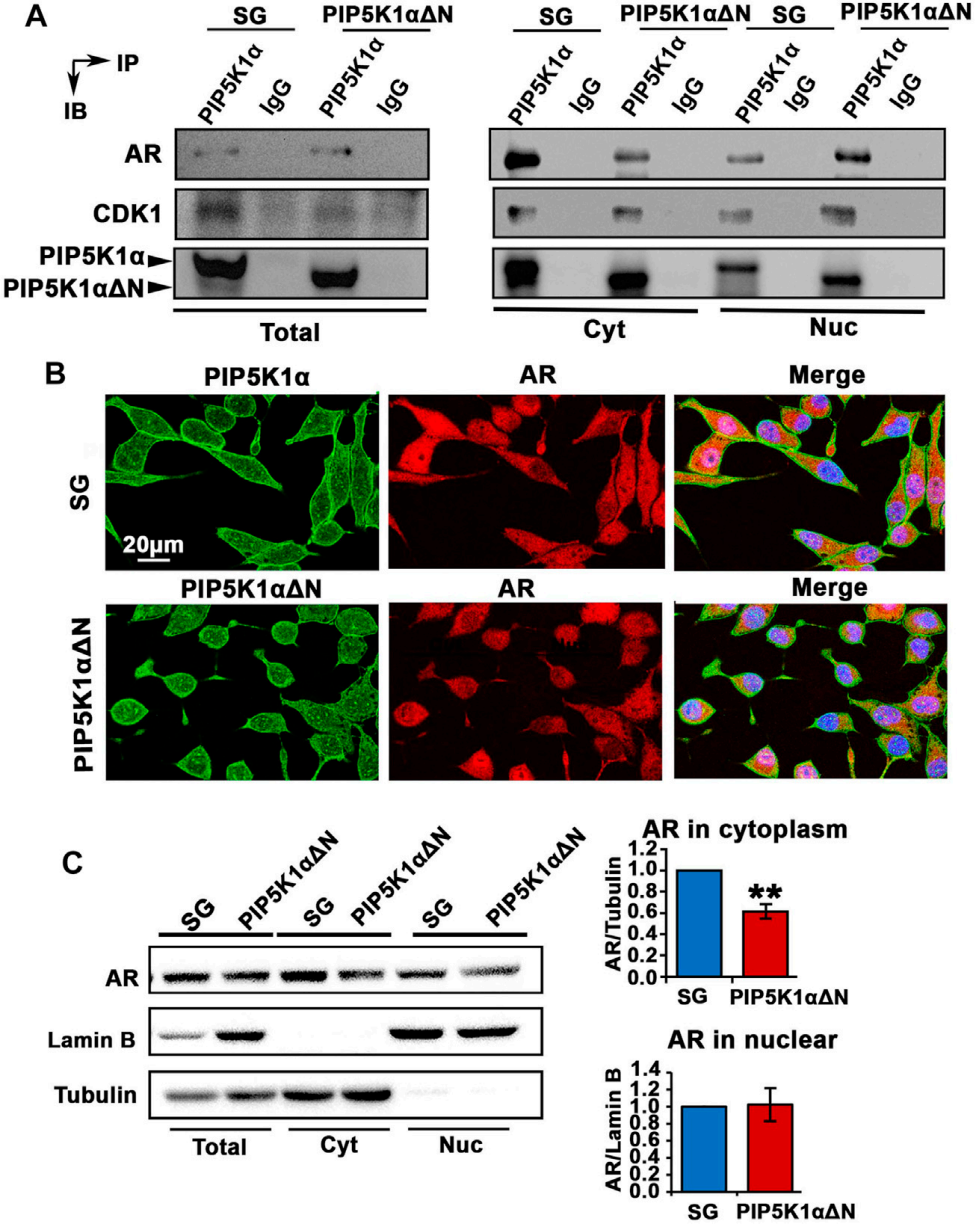
Biological consequences of N-terminal truncated PIP5K1α cells on AR transcriptional activity and AR-associated signalling related to castration resistance. **(A)** Immunoprecipitation assay was performed to examine the formation of protein-protein complexes between PIP5K1α/ΔPIP5K1α and AR in total cell lysates (Total), cytoplasmic (Cyt) and nuclear (Nuc) subcellular fractions of SG cells and PIP5K1αΔN cells. The protein lysates were subjected to the immunoprecipitation (IP) assay. Antibody against PIP5K1α was used to pull down and antibody to IgG was used as a negative control. Antibodies against AR, CDK1 and PIP5K1α were used for immunoblot analysis (IB). The formation of complexes between PIP5K1α/ proteins and AR or CDK1 in different cellular compartments are shown. **(B)** Representative immunofluorescent images show the subcellular localization of AR in red and PIP5K1α proteins in green in SG cells and PIP5K1αΔN cells. Scale bar: 20 μm. **(C)** Immunoblot analysis of AR expression in total cell lysates, cytoplasmic and nuclear compartments of SG and PIP5K1αΔN cells are shown. Tubulin was used as the marker for cytoplasmic fraction, while Lamin B was used as a marker for the nuclear fraction. Quantifications of the immunoblots of AR in subcellular compartments are shown. Data in this figure is presented as the average of three independent experiments (±SE). Student’s *t*-test was used in the analysis. **p < 0.01 is indicated.

Next, we examined whether PIP5K1α serves as co-factor for AR and whether the N-terminal region is essential for PIP5K1α to mediate interactions between PIP5K1α and AR in CRPC cells. To address this, immunoprecipitation assays were performed to assess whether the WT and truncated PIP5K1α proteins may be capable of forming protein-protein complexes with AR. We found that both WT PIP5K1α and truncated PIP5K1α proteins formed protein-protein complexes with AR in the nuclear and cytoplasmic compartments of C4-2 PIP5K1αΔΝ cells and SG control cells (Figure 7A).

We have previously shown that the cyclin dependent kinase CDK1 is a downstream factor of PIP5K1α, which can interact with PIP5K1α through formation of protein-complexes (Sarwar et al., 2016). Moreover, CDK1 is one of the key factors that mediate the stability of AR protein (Chen et al., 2006). We, therefore, examined whether the protein complex between CDK1 and PIP5K1α might be disrupted in PIP5K1αΔΝ cells. Similar to what was observed in SG cells, the formation of complexes between PIP5K1αΔΝ and CDK1 was present in total cells and in the cytoplasmic and nuclear fractions prepared from PIP5K1αΔΝ cells (Figure 7A). This suggests that PIP5K1αΔΝ is able to form complexes with AR and CDK1, indicating that the N-terminal domain is dispensable for PIP5K1α to interact with these partner proteins.

Immunofluorescence staining of PIP5K1α and AR proteins was performed. AR expression in the truncated PIP51K1α cells is mostly nuclear as compared to the SG control cells which have both nuclear and cytoplasmic AR expression (Figure 7B). These data indicate that the N-terminal sequence is required for the cytoplasmic localization of AR. To further assess whether loss of the N-terminus of PIP5K1α would affect the subcellular localization of AR in C4-2 PIP5K1αΔΝ cells and SG control cells, we performed immunoblot analysis. We analyzed AR expression in total cell lysates, cytoplasmic and nuclear compartments of SG and PIP5K1αΔΝ cells, by using Lamin B as a marker for the nuclear fraction and Tubulin for the cytoplasmic fraction (Figure 7C). These resulst confirmed that the cytoplasmic AR expression was significantly decreased in PIP5K1αΔΝ cells compared with that of SG cells (for SG cells, mean cytoplasmic AR = 2.31, 95% CI: 1.83-2.79; for PIP5K1αΔΝ cells, mean cytoplasmic AR = 1.44, difference = 0.86, 95% CI: 0.83-2.06, *p* = 0.0058, Figure 7C). This indicates that PIP5K1α interacts with AR in both the nuclear and cytoplasmic compartments, and although the N-terminal sequence of PIP5K1α may not be essential for association with AR, it appears to play a role in the cytoplasmic localization of AR.

### The N-Terminus of PIP5K1αΔN Is Required for *in vivo* CRPC Tumor Growth

**FIGURE 8:**
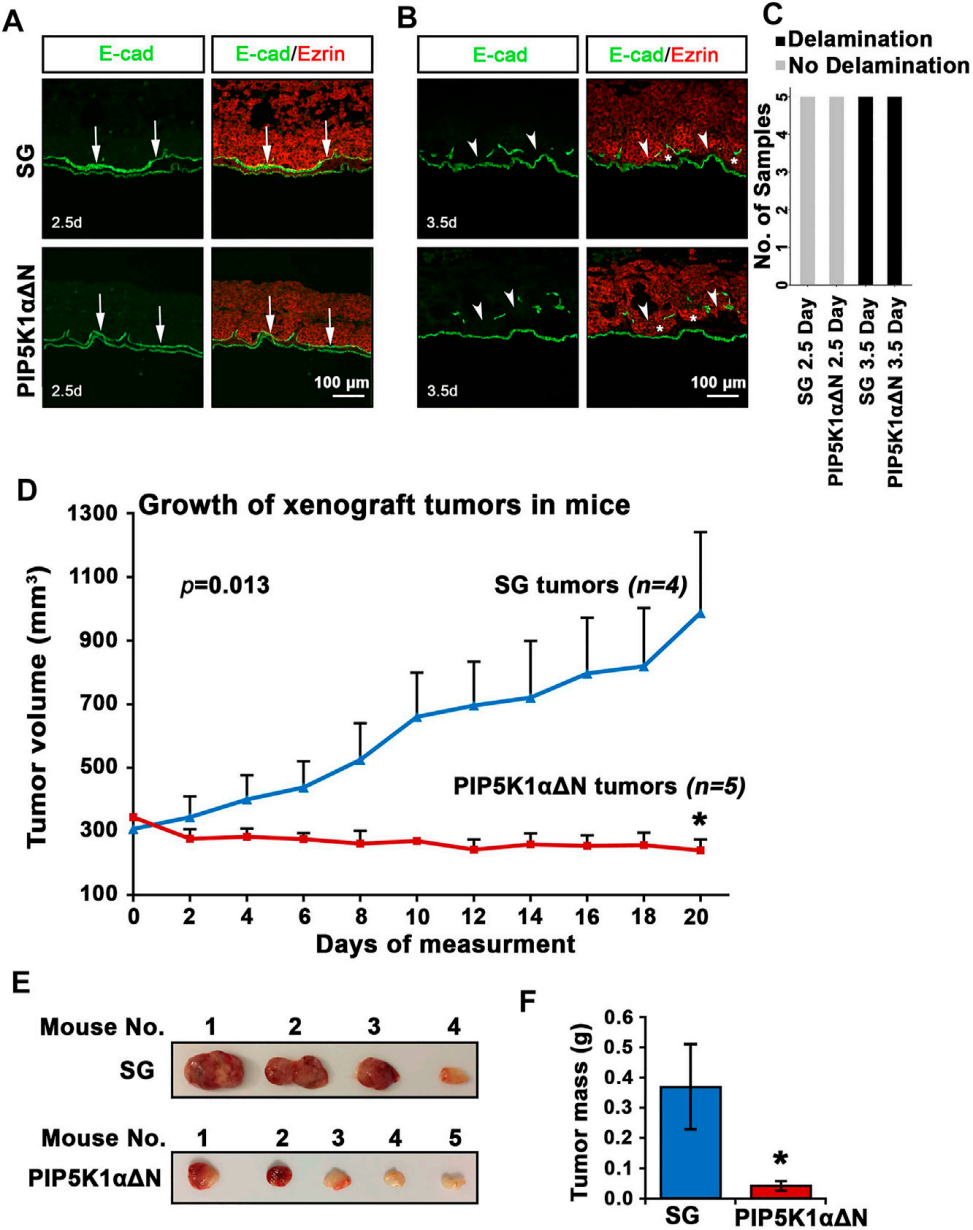
Biological consequences of N-terminal truncated PIP5K1α cells on delamination, initial infiltration, tumor growth and progression. **(A–C)** SG cells and PIP5K1αΔN cells cultured for 2.5 and 3.5 days in the CAM-Delam assay. SG and PIP5K1αΔN cells are identified by Ezrin (red) and the chick basal lamina by laminin-producing E-cadherin positive cells (green). **(A)** At 2.5 days, no delamination or cell infiltration was detected (*n* = 5). **(B)** After 3.5 days, both SG and PIP5K1αΔN cells cause disruption of the basal lamina and cell infiltration into the mesenchyme (*n* = 5). Arrows indicate intact basal lamina **(A)** and disrupted basal lamina **(B)**, and asterisks indicate SG and PIP5K1αΔN infiltrated cells Abbreviation d: days. Scale bar: 100 μm. A bar graph of the CAM-Delam scoring **(C)**. **(D)** Xenograft tumors from SG cells and PIP5K1αΔN cells were established in nude mice. The measurement of tumor started when tumor grew steadily after inoculation. The tumors were measured every second day for 20 days. Mean tumor volumes are shown. **(E)** Representative images of tumors from each group are shown. **(F)** Mean tumor mass in weight from xenograft mice bearing SG tumors and PIP5K1αΔN tumors are shown. Student’s *t*-test is used. *p < 0.05 is indicated.

To further assess biological effects of the deletion of the N-terminus of PIP5K1α on tumor growth and on the initial stage of tumor cell infiltration at the primary tumor site, we first compared the capacity of PIP5K1αΔΝ and SG cells to degrade basal lamina by using the CAM-Delam assay (Palaniappan et al., 2020). To determine the delamination and infiltration capacity, the PIP5K1αΔΝ and SG cells were identified using an Ezrin antibody, and the basal lamina was indirectly defined by E-cadherin, which is expressed in laminin-producing epithelial cells of the chick CAM (Palaniappan et al., 2020). Large disruptions of E-cadherin-positive basement membrane by the infiltrated cancer cells were scored as the effect of delamination, whereas minor disruptions of E-cadherin/basement membrane without infiltrated cancer cells were scored as no delamination effect. After 2.5 days of culture, no major disruptions in the basal lamina were induced by SG or PIP5K1αΔΝ cells as determined by intact E-cadherin expression (Figures 8A,B). In contrast, after 3.5 days, these two types of CRPC cells induced clear E-cadherin-positive membrane disruptions, indicative of delamination, and initiated infiltration into the chick CAM mesenchyme (Figures 8B,C). These results indicate that CRPC C4-2 expressing WT PIP5K1α and truncated PIP5K1α have the capacity to disrupt the basal lamina at the initial stage of infiltration into the basement membranes.

To test the biological consequences of deletion of the N-terminus of PIP5K1α on tumor growth *in vivo*, we employed the xenograft mouse model. PIP5K1αΔΝ cells and SG cells were inoculated subcutaneously into nude mice. The xenograft tumors were established by using Matrigel to support tumor growth to reach at mean tumor volume at approximately 300 mm^3^. We then measured and followed tumor growth every second day for 20 days before ending the experiment. Interestingly, the SG control xenograft tumors grew exponentially faster, and reached a mean volume of 986.17 mm^3^. In contrast, the mean volume of PIP5K1αΔΝ xenograft tumors was only 240.99 mm^3^, 3-fold smaller than that of control SG xenograft tumors, and even lower than their size at the starting point (*p* = 0.0134, Figure 8D). The majority of PIP5K1αΔΝ tumors exhibited a pale colour on their surface (Figure 8E). The mean tumor mass of PIP5K1αΔΝ tumors was also approximately 3-fold less than that of SG control tumors (mean PIP5K1αΔΝ tumor mass was 0.04 g and mean SG control tumor mass was 0.37g, *p* = 0.0336, Figure 8F). These results suggest that deletion of the N-terminus of PIP5K1α significantly reduces the ability of PCa cells to grow and progress to invasive CRPC tumors in mice.

### Deletion of the N-Terminus of PIP5K1α and ISA-2011B-Induced Inhibition in CRPC Cells

**FIGURE 9:**
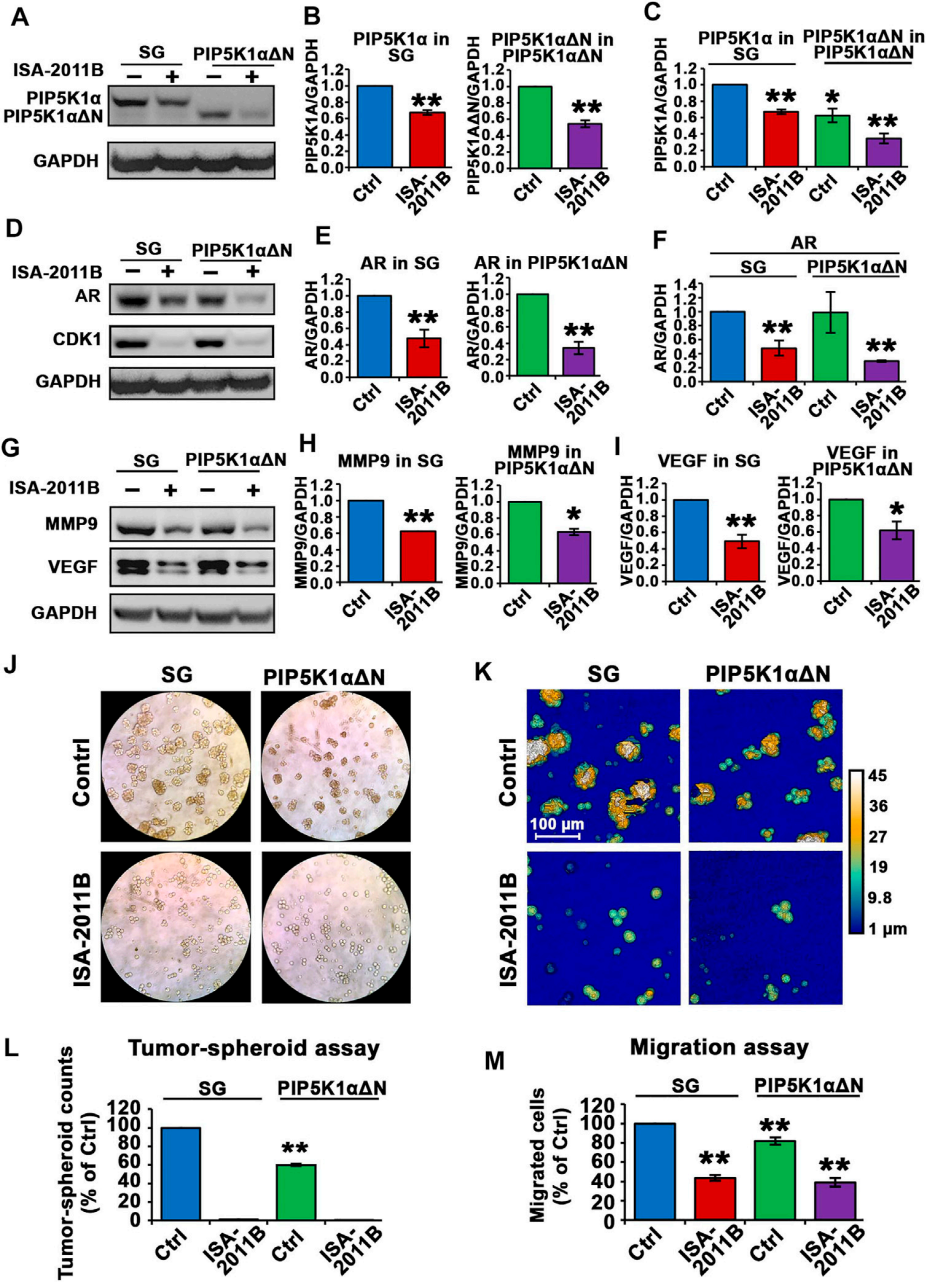
The effect of ISA-2011B on the downstream effectors of PIP5K1α and growth in SG and PIP5K1αΔN cells. **(A–F)** The effect of ISA-2011B on the expression of PIP5K1α, AR and CDK1 in PIP5K1αΔN cells and SG cells was determined using immunoblot analysis. Quantifications of the immunoblots of PIP5K1α and AR in SG or PIP5K1αΔN cells treated with DMSO or ISA-2011B are shown in the right panels. **(G–I)** The effect of ISA-2011B on the expression of MMP9 and VEGF in PIP5K1αΔN cells and SG cells was determined using immunoblot analysis. Quantifications of the immunoblots of the proteins in SG or PIP5K1αΔN cells treated with DMSO or ISA-2011B are shown in the right panels. **(J)** Representative images of the tumor-spheroids derived from SG cells or PIP5K1αΔN cells that were treated with DMSO (Control) or ISA-2011B are shown. **(K)** Representative images of the tumor spheroids were taken using the digital Holomonitor M4. **(L)** The counts of tumor spheroids derived from SG or PIP5K1αΔN cells treated with DMSO or ISA-2011B are shown. **(M)** The effect of ISA-2011B on the migratory ability of SG cells or PIP5K1αΔN cells was determined using migration assays. Data in this figure is presented as the average of three independent experiments (±SE). Student’s *t*-test was used in the analysis. *p < 0.05 or **p < 0.01 are indicated.

Next, we examined whether the effects of ISA-2011B were similar or different between PIP5K1αΔΝ cells and SG control cells. We treated SG control cells and PIP5K1αΔΝ cells with ISA-2011B or DMSO, and ascertained protein expression levels of PIP5K1α. ISA-2011B treatment led to an approximately 50% inhibition of the truncated PIP5K1α in PIP5K1αΔΝ cells (*p <* 0.001, Figures 9A,B). However, only 30% of inhibition on WT PIP5K1α by ISA-2011B was achieved in SG cells (*p <* 0.001, Figures 9A,B). Thus, the effect of ISA-2011B on truncated PIP5K1α was much more pronounced in PIP5K1αΔN cells than it was on the full-length PIP5K1α in SG control cells. When comparing SG cells and PIP5K1αΔΝ cells side-by-side, ISA-2011B treatment led to a distinct reduction in PIP5K1αΔN protein expression by approximately 66% relative to that of full-length PIP5K1α (*p <* 0.001, Figure 9C). Interestingly, ISA-2011B treatment severely diminished AR expression in PIP5K1αΔN cells (*p <* 0.001, Figures 9D–F). Similar to what was observed for AR, ISA-2011B treatment drastically diminished CDK1 expression in SG cells and PIP5K1αΔN cells as well (*p <* 0.001, Figure 9D). In addition, ISA-2011B resulted in a more pronounced inhibitory effect on MMP9 expression in PIP5K1αΔN cells compared with SG control cells (*p <* 0.001; Figures 9G,H). There was also a pronounced inhibitory effect on VEGF expression in PIP5K1αΔN cells as compared with in SG control cells (*p <* 0.001; Figures 9G,I). These data indicate loss of the N-terminus of PIP5K1A is not only important for protein stability, but also that this loss enhances ISA-2011B-induced reductions in the expression of PIP5K1A cofactors AR and CDK1.

To examine the functional consequences of ISA-2011B treatment on SG and PIP5K1αΔN cells, we subjected the cells to the tumor spheroid assays. ISA-2011B treatment completely abrogated the ability of SG control cells and PIP5K1αΔN cells to produce tumor-spheroids (Figures 9J–L,). We next examined the effect of ISA-2011B on migratory ability of SG control cells and PIP5K1αΔN cells. ISA-2011B treatment significantly decreased the migratory ability of SG control cells and PIP5K1αΔN cells as well (For SG vs. PIP5K1αΔN cells, treated with vehicle, *p* = 0.0045; for comparison between other samples, *p <* 0.001, Figure 9M). These findings confirm that PIP5K1α is an intriguing target for cancer treatment, and suggests that the N-terminal domain is an important region for drug targeting.

## Discussion

Expression and activity of AR are highly elevated in castration-resistant PCa to enable cancer cells to gain the advantages of growth, survival and invasion (Takeda et al., 2018). We have previously used castration-resistant PC-3 cells that lack functional AR as the model system to study the role of PIP5K1α. We have shown that selective inhibition of PIP5K1α using its small molecule inhibitor ISA-2011B led to a significantly reduced growth of xenograft PC-3 tumors. However, the precise role and the molecular actions of PIP5K1α in growth and survival of castration-resistant PCa expressing functional AR remained less clear. In this study, we elucidated the role of PIP5K1α in CRPC by using two cell line models: C4-2 and DU145 by using siRNA-mediated knockdown and using a selective inhibitor to silence or inhibit PIP5K1α expression and activity. We demonstrated that siRNA-mediated knockdown of PIP5K1α significantly reduced tumorigenic ability as determined by tumor spheroid assays in both C4-2 cells and DU145 cells. Si-RNA-mediated silencing PIP5K1 resulted a blockade of cell cycle in both C4-2 cells and DU145 cells, which was coincident with significantly decreased expression of AR and CDK1 associated growth and survival pathways in C4-2 and DU145 cells. Moreover, Inhibition of PIP5K1α *via* siRNA-mediated knockdown reduced migratory ability of C4-2 cells and DU145 cells. In agreement with what was achieved by using si-RNA-mediated knockdown of PIP5K1α, inhibition of PIP5K1α using its inhibitor ISA-2011B also resulted in reduced tumorigenic ability and significantly decreased migratory ability, which was co-incident with the downregulation of expression of AR and CDK1 associated pathways in C4-2 cells and DU145 cells. Our findings provided evidence suggesting that PIP5K1α plays an important role in growth and invasion of PCa cells. Our data suggest that PIP5K1α predominantly acts on AR and CDK1-associated cellular pathways to promote growth and invasion of C4-2 and DU145 cells.

In this study, we investigated the functional aspects of PIP5K1α in tumor progression and in mediating the response of CRPC cells to PIP5K1α-inhibitor treatment. We established and employed PIP5K1αΔN C4-2 cells, a CRISPR-edited CRPC cell line that expressed an N-terminally truncated PIP5K1α, without the presence of wild-type PIP5K1α. C4-2 PIP5K1αΔN cells express exclusively truncated PIP5K1α encoded by the mRNA lacking the first ATG fragment, leaving the second ATG intact. Thus, the targeted deletion allowed the production of a truncated PIP5K1α protein and further assessment of the tumorigenic role of PIP5K1α. Interestingly, deletion of the N-terminal sequence of PIP5K1α led to a significant decrease in mRNA and protein expression levels. Moreover, deletion of the N-terminal sequence of PIP5K1α also led to disruption in protein stability. This suggests that the N-terminal region is important for the regulation of PIP5K1α mRNA and protein expression as well as protein stability.

Our results show that full-length PIP5K1α formed protein-protein complexes with AR and CDK1. Interestingly, the truncated PIP5K1α protein also formed protein complexes with AR and CDK1. These findings further provide evidence suggesting that PIP5K1A is functionally associated with AR via protein-protein interactions. Another striking finding is that deletion of the N-terminus of PIP5K1α strongly down-regulated AR-associated proteins including CDK1 and MMP9. The effect of the N-terminal deletion of PIP5K1α was similar to what was achieved via siRNA-knockdown or using its inhibitor ISA-2011B. CDK1 is a key cell cycle regulator that is required for cell proliferation, and it is also functionally associated with AR by mediating AR protein stability (Chen et al., 2006). As a key proteolytic enzyme, MMP9 produced by cancer cells and extracellular matrixes (ECMs) surrounding the tumor can activate angiogenic factors such as VEGF, leading to increased angiogenesis and metastatic activities (Coussens et al., 2000; Giraudo et al., 2004; Overall and Kleifeld, 2006). Moreover, elevated expression of MMP9 is observed in PCa cells in primary and metastatic tissues (Larsson et al., 2020). MMP9 is able to trigger intracellular signaling function to promote proliferation of cancer cells (Khokha et al., 2013; Pego et al., 2018; Larsson et al., 2020). Although our findings in this study support the role of PIP5K1α in angiogenesis and invasion. However, the precise role of PIP5K1α in angiogenesis and cancer cell survivals will be systematically investigated in the near future by using *in vitro* and *in vivo* models to mimic neoangiogenic process.

In respect to tumor aggressiveness, our results show that both PIP5K1αΔΝ and SG cells can trigger basement membrane disruption at the initial stage of infiltration, by using our newly developed *in vivo* chick based CAM-Delam method (Palaniappan et al., 2020), indicating that the N-terminal domain of PIP5K1α is not essential for PCa cells to disrupt the basal lamina at the initial stage of the growth and infiltration. This data is in agreement with what was observed in the xenograft mouse model, in which both PIP5K1αΔΝ and SG cells were capable of establishing tumors in xenograft mice after inoculation. However, CRPC tumors lacking the N-terminal domain of PIP5K1α were unable to progress to aggressive tumors and displayed loss of tumor vascularization, in contrast to that of controls in xenograft mice. This suggests that the reduced growth of xenograft PIP5K1αΔN tumors in mice is a result of loss of oncogenic activity of the truncated PIP5K1α, beyond delamination capacity. Consistently, only SG tumors were able to progress to larger and aggressive tumors in xenograft mice. Mechanistically, PIP5K1αΔN cells express a significantly lower level of the truncated PIP5K1α protein and display decreased expression of AR and CDK1, resulting in reduced growth and survival ability as compared to that of PIP5K1α-WT tumors *in vitro* and in *in vivo*. In this study, we have collected the tumors for further processing and assessment of the biological changes resulted from the PIP5K1a inhibition in this *in vivo* model*.* However, due to that the most of PIP5K1αΔN tumors are tiny in size and no cancer cells were found, there it was not possible for conducting molecular analysis by using the tumor tissues from this *in vivo* model. Nevertheless, in our previous reported studies, we have shown that overexpression of PIP5K1α promotes tumor growth and invasiveness in mouse xenograft models (Sarwar et al., 2016; Sarwar et al., 2019).

In summary, we have shown a clear inhibitory effect of ISA-2011B on AR expression in CRPC cells. Further, ISA-2011B treatment clearly diminished AR expression in PIP5K1αΔN cells. These results indicate that the growth of PIP5K1α-dependent tumors is in part dependent on the integrity of the N-terminal sequence of this kinase. Our new finding identifies the N-terminal domain of the lipid kinase PIP5K1α is important to control the growth of PCa, thus may serve as an ideal drug target for designing new PIP5K1α inhibitor to suppress tumor growth by blocking PIP5K1α activity. Taken together, our study identifies a novel functional mechanism involving PIP5K1α, confirming that PIP5K1α is an intriguing target for cancer treatment, especially for treatment of CRPC. Our findings provide new information to guide the targeted therapy for treatment of invasive castration-resistant prostate cancer.

## Data Availability

The original contributions presented in the study are included in the article, further inquiries can be directed to the corresponding author.

## Abbreviations

PCa, Prostate cancer; CRPC, castration-resistant prostate cancer; PIP5K1α, phosphatidylinositol 4-phosphate 5 kinase; MMP9, matrix metalloproteinases 9; AR, androgen receptor.
